# Informed decisions about public health and social measures

**DOI:** 10.1186/s12961-025-01424-7

**Published:** 2025-11-20

**Authors:** Andrew D. Oxman, Annlaug Selstø, Arnfinn Helleve, Atle Fretheim, Cathinka Halle Julin, Christine Holst, Christopher James Rose, Heather Munthe-Kaas, Ingeborg Hess Elgersma, Jenny Moberg, Mona Bjørbæk, Petter Elstrøm, Runar Barstad Solberg, Sarah E. Rosenbaum, Signe Flottorp, Tone Bruun, Unni Gopinathan

**Affiliations:** Centre for Epidemic Interventions Research, Norwegian Institute of Public Health, Postboks 222, 0213 Skøyen, Oslo Norway

**Keywords:** Public health and social measures, Health crises, Pandemic preparedness, Health communication, Health literacy

## Abstract

Evidence, communication, critical thinking and participation are the cornerstones of informed decisions. In this article we discuss each of these in relation to decisions about public health and social measures (PHSM) during the coronavirus disease 2019 (COVID-19) pandemic and implications for future research. Reliable research evidence of the effects of interventions is particularly important for decisions about what to do because it provides the best basis for estimating the wanted and unwanted effects of doing something. There was little reliable research of the effects of PHSM during the pandemic. For research evidence to be useful to decision-makers, it must be effectively communicated, including how sure we can be about effects or other research findings. Research evidence is essential for making informed decisions, but it is not sufficient. Decision-makers and those affected by the decision must be able to think critically about what to believe and what to do. Many people lack competences and dispositions for thinking critically about PHSM or other interventions. Judgements about PHSM require democratic input, not just expert input. However, there was little public participation in deliberative or decision-making processes about PHSM during the pandemic. There are important uncertainties about the effects of PHSM, how to effectively communicate decisions and evidence about PHSM, how to foster critical thinking about PHSM and how to effectively engage the public in deliberative and decision-making processes about PHSM. Pandemic research and preparedness planning should address those uncertainties.

## Introduction

During the coronavirus disease 2019 (COVID-19) pandemic, public health authorities and governments made many decisions about public health and social measures (PHSM) to reduce transmission of COVID-19 infections and its consequences. This included decisions about testing practices, isolation, quarantining, contact tracing, social distancing, hand washing, facemasks, travel restrictions and closure of schools and businesses. These decisions included both recommendations and mandates. They affected people’s lives in multiple ways. Although they potentially reduced the burden of COVID-19 infections, they also had undesirable health, social and economic consequences. Individual citizens also made decisions about whether to adhere to recommendations and mandates.

One would hope that public health authorities, governments and individuals made informed decisions. Informed decisions do not guarantee that the desirable impacts of a decision will outweigh the undesirable impacts. Many other factors can affect both the decisions that are made and the impacts of those decisions, including political, social and cultural factors. Nonetheless, compared with poorly informed decisions, informed decisions can increase the likelihood that desirable impacts will outweigh undesirable impacts.

**Fig. 1:**
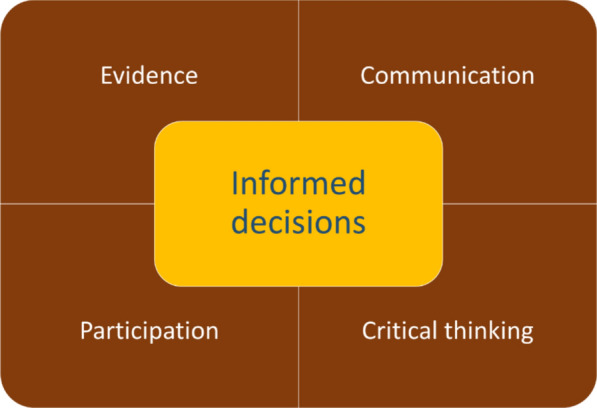
The cornerstones of informed decisions

Informed decisions use the best available information when determining what to do. They give special attention to research evidence. Appropriate use of research evidence depends on it being effectively communicated to decision makers by researchers or others. It also depends on decision-makers’ ability to think critically. Informed decisions about PHSM may also depend on effective public participation. Evidence (particularly research evidence), communication, critical thinking and participation are the cornerstones of informed decision-making (Fig. 1). In this article we will discuss each of these in relation to the COVID-19 pandemic and implications for future research.

## Research evidence

Well-conducted research uses carefully planned and explicit methods to reduce the risk of being misled by bias (systematic errors) and by the play of chance (random errors). Although the importance of research may seem obvious, many people do not trust research evidence. For example, a survey in the United Kingdom found that about two thirds of British adults stated that the experiences of their friends and family were a trustworthy source of information when deciding whether to take or refuse a medicine, whereas only about one third trusted evidence from medical trials [1].

Evaluating the effects of interventions depends on comparisons, for example, between a group of people that is exposed to an intervention and a group that is not. If the people being compared differ in ways other than the interventions being compared, the apparent effects of the interventions might reflect those differences rather than intervention effects. The value of randomized trials is that they control for both measured and unmeasured characteristics. This results in comparison groups that are similar in terms of prognostic variables, whether these have been recognized or not. This does not, however, guarantee that the results of randomized trials are unbiased. Both randomized trials and nonrandomized studies can be misleading for other reasons [2].

### An inspiring response to the pandemic

The need for evidence to inform decisions about pharmacological interventions for COVID-19 was addressed by thousands of randomized trials of vaccines and drugs that identified both effective and ineffective interventions. This response to the pandemic was inspiring, sometimes disappointing and often frightening. It was inspiring because of the rapid response and gains made by appropriate use of research. At the start of the pandemic, little was known about the effects of vaccines and drugs. However, in less than a year, over 2000 randomized trials were registered [3].

### A disappointing response to the pandemic

**Fig. 2:**
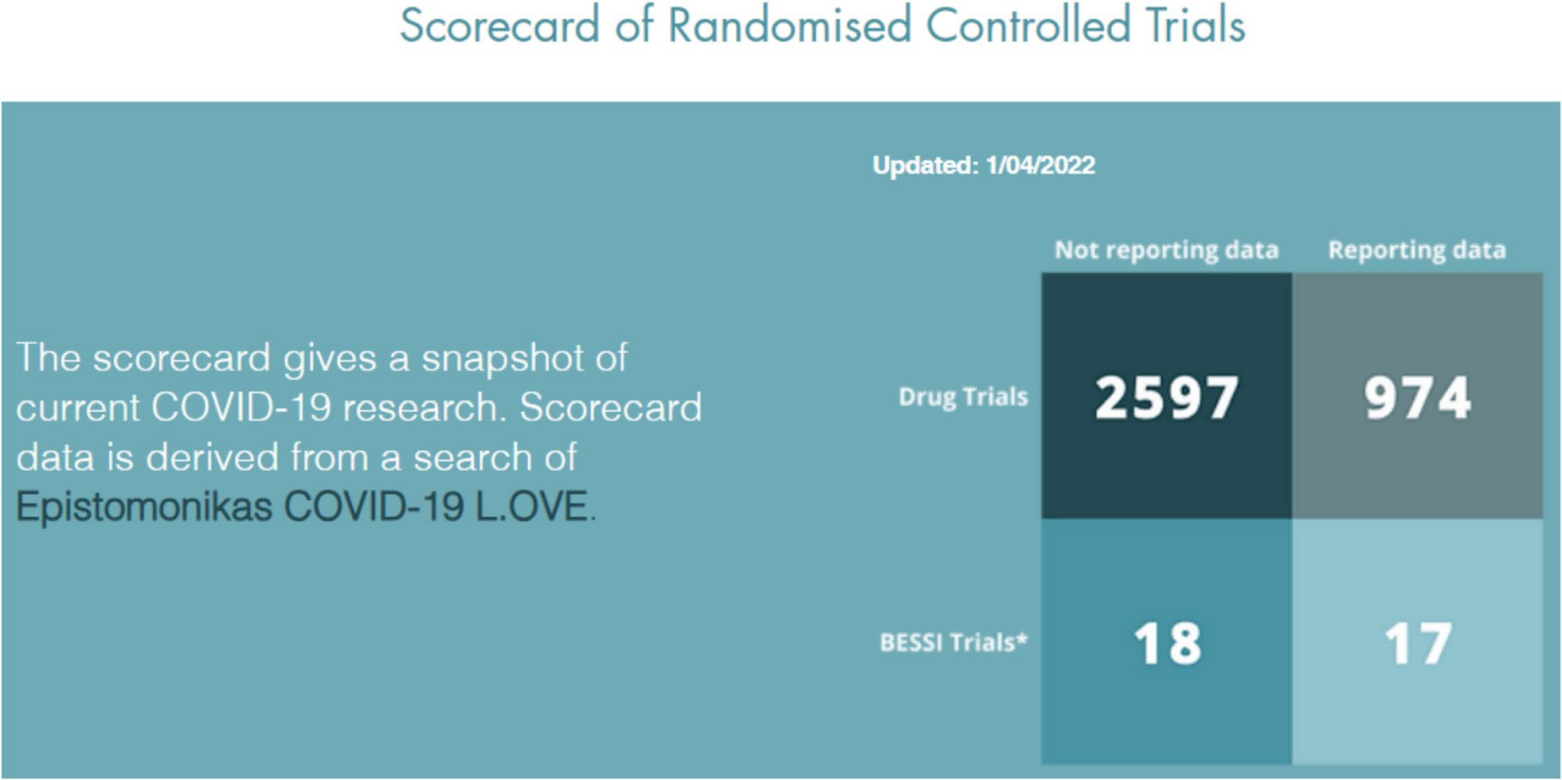
Pharmacological and nonpharmacological (BESSI) trials^*^. ^*^Behavioural, Environmental, Social and Systems Interventions (BESSI) https://www.bessi-collab.net/

The response to the pandemic was disappointing for three reasons. First, many drug trials were too small to reliably inform decisions and were not coordinated. Second, people sometimes did not use or benefit from research. Third, there were inadequate efforts to address important questions about nonpharmacological interventions. As of April 2022, a total of 3571 COVID-19 drug trials had been registered and 974 of those had reported results. Meanwhile, only 35 trials of nonpharmacological interventions had been registered and 17 of those had reported results (Fig. 2). There have been very few reports of reliable evaluations of the effects of COVID-19 policies (such as stay-at-home requirements and closing of schools) [4], and there are major uncertainties about the effects – wanted and unwanted – of those policies.

Estimates of the effects of policies such as closing schools were often based on modelling studies (numerical simulations of how different factors might influence the course of the pandemic) and nonrandomized studies. Modelling studies frequently rely on a range of assumptions, often resulting in outcomes that can vary when these assumptions are changed. For example, some modelling studies suggested that school closures could reduce community transmission of the coronavirus [5], while others disagreed [6]. These conflicting results may be due to different assumptions about, among other things, per-contact transmission probabilities, changes in contacts outside of home owing to schools closing or opening, and what other protective measures are in place. Because of assumptions such as these and important uncertainty about many of them, the results of these modelling studies were very uncertain.

Early in the pandemic, some assumptions were empirically informed, such as how populations are distributed spatially. However, there was limited, if any, empirical evidence for other assumptions, such as an assumption that children were twice as likely as adults to transmit the coronavirus. That assumption helped justify school closures. However, subsequent epidemiological studies suggested little or no difference in transmission of the virus from children compared with adults in households and low secondary attack rates in schools [7, 8]. Modelling studies of the effects of interventions can be helpful, but it is important to recognize their limitations.

### A frightening response to the pandemic

The response to the pandemic was frightening because of the sometimes overwhelming amount of misleading information and the impacts that had on people’s lives [9], including research with important limitations that was frantically hyped as certain [10]. Critical appraisal of research by systematic review authors can help reduce the likelihood of being misled by hyped research. High-quality systematic reviews are important for the same reason that high-quality research is important – to reduce the risk of being misled by bias and the play of chance. Systematic reviews use carefully planned and explicit methods to identify, select and critically appraise relevant studies and to collect and analyse data from them.

In the context of the pandemic, reviews had to be carried out quickly as well as systematically. There was an explosion of rapid systematic reviews on all aspects of COVID-19, including reviews on the effects of PHSM. However, many reviews described as systematic had important shortcomings, including not critically appraising the included studies [11]. There also was unnecessary duplication of efforts. The quality of systematic reviews and ensuring that they are up to date could be improved by including the need for rapid and reliable systematic reviews in pandemic preparedness planning and use of machine learning and artificial intelligence technologies [12]. Better international coordination of efforts could help to reduce unnecessary duplication of efforts.

### Evidence does not make decisions

When deciding what to do, many types of evidence are important. This includes evidence of

- The baseline risk or severity of the problem – for example, whether there are few cases of COVID-19 or a surge of cases
- How important the wanted and unwanted outcomes are – for example, wanted outcomes such as a reduction in the spread of infections and people with serious illness and unwanted outcomes of school closures such as lost opportunities to learn and socialize
- Economic consequences
- Impacts on equity
- The acceptability and feasibility of interventions

Reliable evidence of the effects of interventions is particularly important for decisions about what to do because it provides the best basis for estimating the wanted and unwanted effects of doing something.

Research evidence is essential for making informed decisions, but it is not sufficient. Neither policy decisions nor personal choices are determined by evidence alone. Judgements, values and other factors always play a role [13]. Claims that COVID-19 policies did or should “follow the science” obscure important aspects of policy-making and how decisions were made and should be made [14].

## Communication

To be useful, research evidence must be accessible and understandable to the people making decisions. This includes public health authorities, governments and individuals making personal choices. Evidence-based information about the effects of interventions should use language that is appropriate for the audience, and it should communicate the certainty of the evidence [15].

Simply relying on expert advice without knowing the basis for the advice is not sufficient and can be misleading. Experts may not base their opinions on systematic reviews of the best available evidence, and they may not be aware of all the relevant research [16]. Their opinions may be affected by cognitive heuristics and bias [17]. They may have conflicts of interest, and they frequently disagree.

Messages from public health authorities and governments about PHSM changed as the pandemic evolved [18]. Justifications for these changes were not always shared candidly in communication by health authorities or governments [19]. As a result, people sometimes experienced COVID-19 messages from public health authorities and governments as untruthful and inconsistent. Thus, those messages may have exacerbated rather than reduced confusion from the infodemic that accompanied the pandemic [9]. Transparent communication of the evidence, including how sure we can be, does not undermine public trust and, in fact, may increase the perceived trustworthiness of public health messages, trust in science and trust in public health authorities and governments [20, 21].

Systematic procedures should be used to help ensure that decisions about recommendations and policies and about how to communicate those decisions are informed by evidence [22]. Systematic procedures and transparency do not guarantee reasonable decisions, but they can help to ensure understanding, accountability and reasonableness [23].

When there is a public health emergency such as a pandemic, policies that require certain behaviours, such as staying at home, may be justified despite important uncertainties about the balance between the potential benefits and harms [22]. However, when there are important uncertainties, they should be acknowledged. Not disclosing uncertainties distorts what is known, inhibits research to reduce important uncertainties and can undermine public trust in health authorities [19–22, 24]. When there are important uncertainties about the effects of PHSM, including how to communicate uncertainty, they should be evaluated – if possible, in randomized trials. Similarly, important uncertainties about how best to communicate decisions or evidence of the effects of PHSM should be evaluated – for example, by user testing messages and in online randomized trials assessing the effects of messages on people’s understanding of the messages, decisions and intended behaviours [20–22, 25].

## Critical thinking

For decisions to be informed by research evidence and for the basis for decisions to be understood and trusted, decision-makers and those affected by the decision must be able to think critically about what to believe and what to do. To think critically about the effects of interventions, people need to be able to understand and apply basic concepts (principles) for assessing the reliability of claims about effects, assessing evidence supporting those claims and making informed decisions [26]. Unfortunately, many people neither understand nor apply many of these concepts [27].

Critical thinking is consistently associated with reduced susceptibility to misinformation, both in general and specifically about COVID-19 [28]; and susceptibility to misinformation is associated with vaccine hesitancy and a reduced likelihood of complying with public health guidance [28, 29]. Thus, fostering critical thinking skills can both reduce susceptibility to misinformation and increase the likelihood that reliable information will be recognized and used appropriately [30].

Systematic reviews have found limited evidence of the effectiveness of interventions to teach motivated adults generic critical thinking skills [31] and critical thinking about health interventions specifically [32]. However, many, if not most, adults are unlikely to be motivated owing to barriers to adult education and barriers to fostering critical thinking in adults. Barriers to adult education include lack of time, other responsibilities, lack of confidence and dislike of studying [33]. Barriers to fostering critical thinking in adults include prejudices and values that have been instilled in us by our culture, psychological coping strategies that distort reality to protect ourselves from bad feelings and biases in our thinking and perception that serve to protect or elevate our self-esteem [34].

Teaching children and young people to think critically about interventions may be more promising than teaching adults [35, 36]. By targeting school children, it is possible to reach a large segment of the population before many leave the education system and become difficult to reach. Teaching school children can capitalize on the time they have available for learning and their motivation to learn, and it can lay a foundation for them to think critically as adults.

## Participation

During the pandemic, public health authorities and governments made difficult decisions about PHSM. Decisions needed to be made quickly, with limited or uncertain evidence of the effects of those measures and important trade-offs between the potential benefits and harms. Although nearly everyone was affected by at least some of these decisions, there appears to have been little public participation in deliberative or decision-making processes about PHSM [37, 38]. One reason for this is that participation takes time, especially if it is not already institutionalized [39], and decisions needed to be made quickly.

The justification for actions taken by governments and public health authorities, their empowerment, and the lack of participation is embedded in the precautionary principle: In response to urgent and credible threats of serious harm, proportionate precautions should be taken. This is a complex principle that requires judgements about the urgency of a threat, the credibility of the threat, the likelihood and seriousness of the potential harms, and the potential benefits and harms of interventions [40]. Those judgments require democratic input, not just expert input [41]. In addition, those judgements should be informed by the best available evidence, and application of the precautionary principle should include scientific evaluation to both highlight and reduce important uncertainties when evidence is lacking [42].

As noted in the Alma-Ata Declaration from the International Conference on Primary Health Care, “The people have the right and duty to participate individually and collectively in the planning and implementation of their health care” [43]. In addition to being a democratic right, participation in deliberative and decision-making processes has the potential to improve the quality of the judgements and decisions that are made, build trust, improve adherence and help to ensure transparency and accountability [41]. Engaging members of the public can help to ensure that:

- Their concerns are heard and considered.
- Marginalized communities are included in decision-making.
- Problems are analysed, described and perceived correctly.
- Appropriate solutions are identified.
- Important barriers to implementing solutions are considered.
- Effective implementation strategies are identified.
- Appropriate values are used when weighing the pros and cons of options.
- Policy decisions are appropriate, understood and acceptable.
- Important uncertainties are identified and addressed.

However, public participation may not always be helpful. Poorly planned and implemented participation can create mistrust, waste people’s time and undermine future attempts to engage the public [44]. Participation without clear objectives may anger participants and fail to add benefit to the policy-making process or outcomes. Care should also be taken not to use participation for inappropriate reasons. Sometimes, for example, participation may simply be used to legitimize decisions that have already been made behind closed doors, and people may be misled into believing that they are able to affect the decision. Similarly, public participation should not be used simply to allow authorities to avoid responsibility for difficult decisions.

The extent to which the potential benefits of participation are achieved and potential harms are avoided is likely to depend on several factors, including: inclusive representation (paying special attention to marginalized communities) [45], an appropriate level of participation with clear expectations [46], use of effective methods for participation, use of a systematic and transparent decision-making process [47], and critical thinking.

A review of public participation in decisions about PHSM to control the COVID-19 pandemic found that almost all of the 24 reported initiatives took place in high-income countries, involved consulting the public and consisted of public meetings (usually online) [38]. Only two initiatives reported explicit support for critical thinking. Almost half of the reported initiatives did not contribute to a decision, and 17 initiatives did not include any explicit decision-making criteria.

The reported initiatives point out potential good practice related to online engagement, crowdsourcing and addressing potential power imbalance; and sensible recommendations for engaging the public in decisions about pandemic or other health policies [39, 48]. However, there are important uncertainties about how best to engage the public in decision-making processes. Future research should address improved reporting of public participation initiatives, the use of explicit decision-making criteria, support for critical thinking about the effects of interventions, engagement of marginalized groups, engagement of decision-makers, communication with the broader public and evaluation of both desirable and undesirable impacts of public participation initiatives [38].

## Conclusions

Decisions about PHSM to control the COVID-19 pandemic were often based on unreliable information or evidence with important limitations. This resulted undoubtedly in both wanted and unwanted impacts.

Efforts should be made to reduce the uncertainties about the effects of PHSM measures, as well as about how to best:

- Communicate decisions and the underlying evidence
- Foster clear and rational thinking about what to believe and what to do
- Ensure democratic input into decisions about PHSM

Effective preparedness planning should include research to reduce all those uncertainties.

## Data Availability

No datasets were generated or analysed during the current study.

## References

[1] Academy of Medical Sciences. Enhancing the use of scientific evidence to judge the potential benefits and harms of medicines. London: Academy of Medical Sciences London; 2017.

[2] Sterne JA, Hernán MA, Reeves BC, Savović J, Berkman ND, Viswanathan M, et al. ROBINS-I: a tool for assessing risk of bias in non-randomised studies of interventions. BMJ. 2016;355:i4919. 27733354 10.1136/bmj.i4919 PMC5062054

[3] Dillman A, Zoratti MJ, Park JJH, Hsu G, Dron L, Smith G, et al. The landscape of emerging randomized clinical trial evidence for COVID-19 disease stages: a systematic review of global trial registries. Infect Drug Resist. 2020;13:4577–87. 33376364 10.2147/IDR.S288399 PMC7764888

[4] Haber NA, Clarke-Deelder E, Feller A, Smith ER, Salomon JA, MacCormack-Gelles B, et al. Problems with evidence assessment in COVID-19 health policy impact evaluation: a systematic review of study design and evidence strength. BMJ Open. 2022;12(1):e053820. 35017250 10.1136/bmjopen-2021-053820 PMC8753111

[5] Panovska-Griffiths J, Kerr CC, Stuart RM, Mistry D, Klein DJ, Viner RM, et al. Determining the optimal strategy for reopening schools, the impact of test and trace interventions, and the risk of occurrence of a second COVID-19 epidemic wave in the UK: a modelling study. Lancet Child Adolesc Health. 2020;4(11):817–27. 32758453 10.1016/S2352-4642(20)30250-9 PMC7398659

[6] Davies NG, Kucharski AJ, Eggo RM, Gimma A, Edmunds WJ. Effects of non-pharmaceutical interventions on COVID-19 cases, deaths, and demand for hospital services in the UK: a modelling study. Lancet Public Health. 2020;5(7):e375–85. 32502389 10.1016/S2468-2667(20)30133-X PMC7266572

[7] Viner R, Waddington C, Mytton O, Booy R, Cruz J, Ward J, et al. Transmission of SARS-CoV-2 by children and young people in households and schools: a meta-analysis of population-based and contact-tracing studies. J Infect. 2022;84(3):361–82. 34953911 10.1016/j.jinf.2021.12.026 PMC8694793

[8] Zheng B, Chen H, Xia W, Jiang Y, Zhang J. Secondary infections of COVID-19 in schools and the effectiveness of school-based interventions: a systematic review and meta-analysis. Public Health. 2024;229:42–9. 38394706 10.1016/j.puhe.2024.01.014

[9] Borges do Nascimento IJ, Pizarro AB, Almeida JM, Azzopardi-Muscat N, Gonçalves MA, Björklund M, et al. Infodemics and health misinformation: a systematic review of reviews. Bull World Health Organ. 2022;100(9):544–61. 36062247 10.2471/BLT.21.287654 PMC9421549

[10] Hyland K, Jiang F. The Covid infodemic: competition and the hyping of virus research. Int J Corpus Linguist. 2021;26(4):444–68.

[11] Abbott R, Bethel A, Rogers M, Whear R, Orr N, Shaw L, et al. Characteristics, quality and volume of the first 5 months of the COVID-19 evidence synthesis infodemic: a meta-research study. BMJ Evid-Based Med. 2021. 10.1136/bmjebm-2021-111710. 34083212 10.1136/bmjebm-2021-111710 PMC9132873

[12] Heron L, Buitrago-Garcia D, Ipekci AM, Baumann R, Imeri H, Salanti G, et al. How to update a living systematic review and keep it alive during a pandemic: a practical guide. Syst Rev. 2023;12(1):156. 37660117 10.1186/s13643-023-02325-y PMC10474670

[13] Oxman AD, Lavis JN, Lewin S, Fretheim A. SUPPORT tools for evidence-informed health policymaking (STP) 1: what is evidence-informed policymaking? Health Res Policy Syst. 2009;7(Suppl 1):S1. 20018099 10.1186/1478-4505-7-S1-S1 PMC3271820

[14] Greenmyer JR. “Follow the Science” in COVID-19 policy: a scoping review. *HEC Forum.* 2024. 10.1007/s10730-10024-09521-w. 10.1007/s10730-024-09521-w 38472729

[15] Oxman AD, Glenton C, Flottorp S, Lewin S, Rosenbaum S, Fretheim A. Development of a checklist for people communicating evidence-based information about the effects of healthcare interventions: a mixed methods study. BMJ Open. 2020;10(7):e036348. 32699132 10.1136/bmjopen-2019-036348 PMC7375421

[16] Antman EM, Lau J, Kupelnick B, Mosteller F, Chalmers TC. A comparison of results of meta-analyses of randomized control trials and recommendations of clinical experts. Treatments for myocardial infarction. JAMA. 1992;268(2):240–8. 1535110

[17] Morgan MG. Use (and abuse) of expert elicitation in support of decision making for public policy. Proc Natl Acad Sci U S A. 2014;111(20):7176–84. 24821779 10.1073/pnas.1319946111 PMC4034232

[18] Balog-Way DHP, McComas KA. Covid-19: reflections on trust, tradeoffs, and preparedness. J Risk Research. 2020;23(7–8):838–48.

[19] McCartney M, Sullivan F, Heneghan C. Information and rational decision-making: explanations to patients and citizens about personal risk of Covid-19. BMJ Evid-Based Med. 2020. 10.1136/bmjebm-2020-111541. 33077575 10.1136/bmjebm-2020-111541

[20] Kerr JR, Schneider CR, Freeman AL, Marteau T, van der Linden S. Transparent communication of evidence does not undermine public trust in evidence. PNAS Nexus 2022;1(5):pgac280. 10.1093/pnasnexus/pgac280 PMC9802351 36712327

[21] Batteux E, Bilovich A, Johnson SGB, Tuckett D. Negative consequences of failing to communicate uncertainties during a pandemic: an online randomised controlled trial on COVID-19 vaccines. BMJ Open. 2022;12(9):e051352. 36691187 10.1136/bmjopen-2021-051352 PMC9453426

[22] Oxman AD, Fretheim A, Lewin S, Flottorp S, Glenton C, Helleve A, et al. Health communication in and out of public health emergencies: to persuade or to inform? Health Res Policy Syst. 2022;20:28. 35248064 10.1186/s12961-022-00828-z PMC8897761

[23] Daniels N. Accountability for reasonableness. BMJ. 2000;321(7272):1300–1. 11090498 10.1136/bmj.321.7272.1300 PMC1119050

[24] Dries C, McDowell M, Rebitschek FG, Leuker C. When evidence changes: communicating uncertainty protects against a loss of trust. Public Underst Sci. 2024. 10.1177/09636625241228449. 38414113 10.1177/09636625241228449

[25] Woloshin S, Dewitt B, Krishnamurti T, Fischhoff B. Assessing how consumers interpret and act on results from at-home Covid-19 self-test kits: a randomized clinical trial. JAMA Intern Med. 2022;182(3):332–41. 35099501 10.1001/jamainternmed.2021.8075 PMC8804977

[26] Oxman AD, Chalmers I, Dahlgren A. Key concepts for informed health choices: where’s the evidence? F1000Res. 2023;11:890. 37928808 10.12688/f1000research.123051.2 PMC10623542

[27] Dahlgren A, Furuseth-Olsen K, Rose CJ, Oxman AD. The Norwegian public’s ability to assess treatment claims: results of a cross-sectional study of critical health literacy. F1000Res. 2021;9:179. 38585673 10.12688/f1000research.21902.2 PMC10995534

[28] Roozenbeek J, Schneider CR, Dryhurst S, Kerr J, Freeman ALJ, Recchia G, et al. Susceptibility to misinformation about Covid-19 around the world. R Soc Open Sci. 2020;7(10):201199. 33204475 10.1098/rsos.201199 PMC7657933

[29] Cannito L, Ceccato I, Bortolotti A, Di Crosta A, La Malva P, Palumbo R, Di Domenico A, Palumbo R: Exploring vaccine hesitancy: the twofold role of critical thinking. Curr Psychol 2022;1–9. 10.1007/s12144-022-04165-w PMC9795421 36590014

[30] Swire-Thompson B, Lazer D. Public health and online misinformation: challenges and recommendations. Annu Rev Public Health. 2020;41:433–51. 31874069 10.1146/annurev-publhealth-040119-094127

[31] Abrami PC, Bernard RM, Borokhovski E, Waddington DI, Wade CA, Persson T. Strategies for teaching students to think critically: a meta-analysis. Rev Educ Res. 2015;85(2):275–314.

[32] Cusack L, Del Mar CB, Chalmers I, Gibson E, Hoffmann TC. Educational interventions to improve people’s understanding of key concepts in assessing the effects of health interventions: a systematic review. Syst Rev. 2018;7(1):68. 29716639 10.1186/s13643-018-0719-4 PMC5930693

[33] Morris TA. Anytime/anywhere online learning: does it remove barriers for adult learners? In: Online education and adult learning: new frontiers for teaching practices. Edited by Kidd T. Hershey PA: IGI Global; 2010:115–123.

[34] Kirby GR, Goodpaster JR, Levine M. Critical thinking. Boston, MA: Pearson Custom Publishing; 1999.

[35] Nsangi A, Semakula D, Oxman AD, Austvoll-Dahlgren A, Oxman M, Rosenbaum S, et al. Effects of the informed health choices primary school intervention on the ability of children in Uganda to assess the reliability of claims about treatment effects: a cluster-randomised controlled trial. Lancet. 2017;390(10092):374–88. 28539194 10.1016/S0140-6736(17)31226-6

[36] Chesire F, Mugisha M, Ssenyonga R, Rose CJ, Nsangi A, Kaseje M, et al. Effects of the informed health choices secondary school intervention: a prospective meta-analysis. J Evid Based Med. 2023;16(3):321–31. 37735807 10.1111/jebm.12552

[37] Loewenson R, Colvin CJ, Szabzon F, Das S, Khanna R, Coelho VSP, et al. Beyond command and control: a rapid review of meaningful community-engaged responses to COVID-19. Glob Public Health. 2021;16(8–9):1439–53. 33734007 10.1080/17441692.2021.1900316

[38] Munthe-Kaas H, Oxman AD, von Lieres B, Gloppen S, Ohren A. Public participation in decisions about measures to manage the Covid-19 pandemic: a systematic review. BMJ Glob Health. 2023. 10.1136/bmjgh-2023-014404 PMC11149118 38830748

[39] World Health Organization. Implementing citizen engagement within evidence-informed policy-making: an overview of purpose and methods. Geneva: World Health Organization; 2022.

[40] Weir E, Schabas R, Wilson K, Mackie C. A Canadian framework for applying the precautionary principle to public health issues. Can J Public Health. 2010;101(5):396–8. 21214055 10.1007/BF03404860 PMC6974129

[41] Norheim OF, Abi-Rached JM, Bright LK, Bærøe K, Ferraz OLM, Gloppen S, et al. Difficult trade-offs in response to COVID-19: the case for open and inclusive decision making. Nat Med. 2021;27(1):10–3. 33340033 10.1038/s41591-020-01204-6

[42] Bretthauer M, Helsingen LM, Løberg M, Kalager M, Guyatt G. Evidence and precaution for legal health interventions: learning from the Covid-19 pandemic. Ann Intern Med. 2021;174(10):1456–7. 34370512 10.7326/M21-2839 PMC8372032

[43] World Health Organization. Declaration of Alma-Ata. Geneva: World Health Organization; 1978.

[44] Involve. People & Participation: How to put citizens at the heart of decision-making. London: Involve; 2005.

[45] Dacombe R, Parvin P. Participatory democracy in an age of inequality. Represent. 2021;57(2):145–57.

[46] IAP2 Public Participation Spectrum [https://cdn.ymaws.com/www.iap2.org/resource/resmgr/pillars/Spectrum_8.5x11_Print.pdf]

[47] Moberg J, Oxman AD, Rosenbaum S, Schunemann HJ, Guyatt G, Flottorp S, et al. The GRADE Evidence to Decision (EtD) framework for health system and public health decisions. Health Res Policy Syst. 2018;16(1):45. 29843743 10.1186/s12961-018-0320-2 PMC5975536

[48] Marston C, Renedo A, Miles S. Community participation is crucial in a pandemic. Lancet. 2020;395(10238):1676–8. 32380042 10.1016/S0140-6736(20)31054-0 PMC7198202

